# Phaeohyphomycotic cyst

**DOI:** 10.4322/acr.2023.417

**Published:** 2023-01-11

**Authors:** Rashmi Joshi, Suvradeep Mitra, Mayur Parkhi, Arunanshu Behera

**Affiliations:** 1 Post Graduate Institute of Medical Education and Research, Department of Histopathology, Chandigarh, India; 2 Post Graduate Institute of Medical Education and Research, Department of General Surgery, Chandigarh, India

**Keywords:** Mycoses, Pathology, Melanins, Hand, Farmers

Phaeohyphomycosis is a term used for a rare opportunistic infection caused by a group of dematiaceous fungi which contains melanin in their cell walls. In 1974, the term phaeohyphomycosis was first coined by Ajello for an entity caused by pigmented fungi.^1^ Four clinical forms of phaeohyphomycosis exist: i) cutaneous, ii) subcutaneous, iii) systemic, and iv) cerebral. Among these, the subcutaneous form (phaeohyphomycotic cyst) is the most common subtype which usually presents as nodular swelling mainly over the distal extremities, which may be misdiagnosed as epidermal inclusion cyst, ganglion or lipoma. These fungi are present in the soil, where they infect mostly farmers and persons working in fields and farms. It was also highlighted that these infections are seen mostly in immunocompromised individuals and are byproducts of antimicrobial, steroid, and immunosuppressive therapy for various illnesses, including cancer, autoimmune diseases, and transplant cases.^1^ The pigment giving the characteristic brownish-black appearance to the fungi is melanin, which prevents phagocytosis and hence acts as a virulence factor.^2^ This group has more than 120 species and 70 genera.^3^ They have a broad spectrum of clinical manifestations, including superficial and deep fungal infections, sinus involvement, and disseminated forms, including lung and brain abscesses. The incidence ranges from 1-3.1 per 100,000 patients.^4^ The gold standard method for diagnosis is histopathological examination and culture. Fontana-Masson stain is of immense help in identifying these dematiaceous fungi in tissue as it highlights the melanin pigment in the cell walls.

Figure 1 refers to a 59-year-old farmer man who presented with nodular swelling over the dorsum of the left hand for the last 9 months. Initially, the swelling was of peanut size, which gradually progressed to the present size of 5x3x2cm. The lesion was painless, well-defined, and freely mobile. The joint was not affected. He is on medication for type II diabetes mellitus and hypertension. He underwent Whipple's surgery for periampullary carcinoma. He was treated for proximal sensory-motor axonal neuropathy and tuberculosis three years back. Because of isoniazid-induced hepatitis, the patient received a modified anti-tuberculous regime for 9 months, after which he developed gastric ulceration with hematemesis and melena. On endoscopic biopsy, he was found to have chronic active gastritis with Helicobacter pylori infection. In addition, the patient had severe iron deficiency and hypoalbuminemia due to malabsorption. After treatment, he completely recovered at the time of hospital discharge.

**Figure 1:**
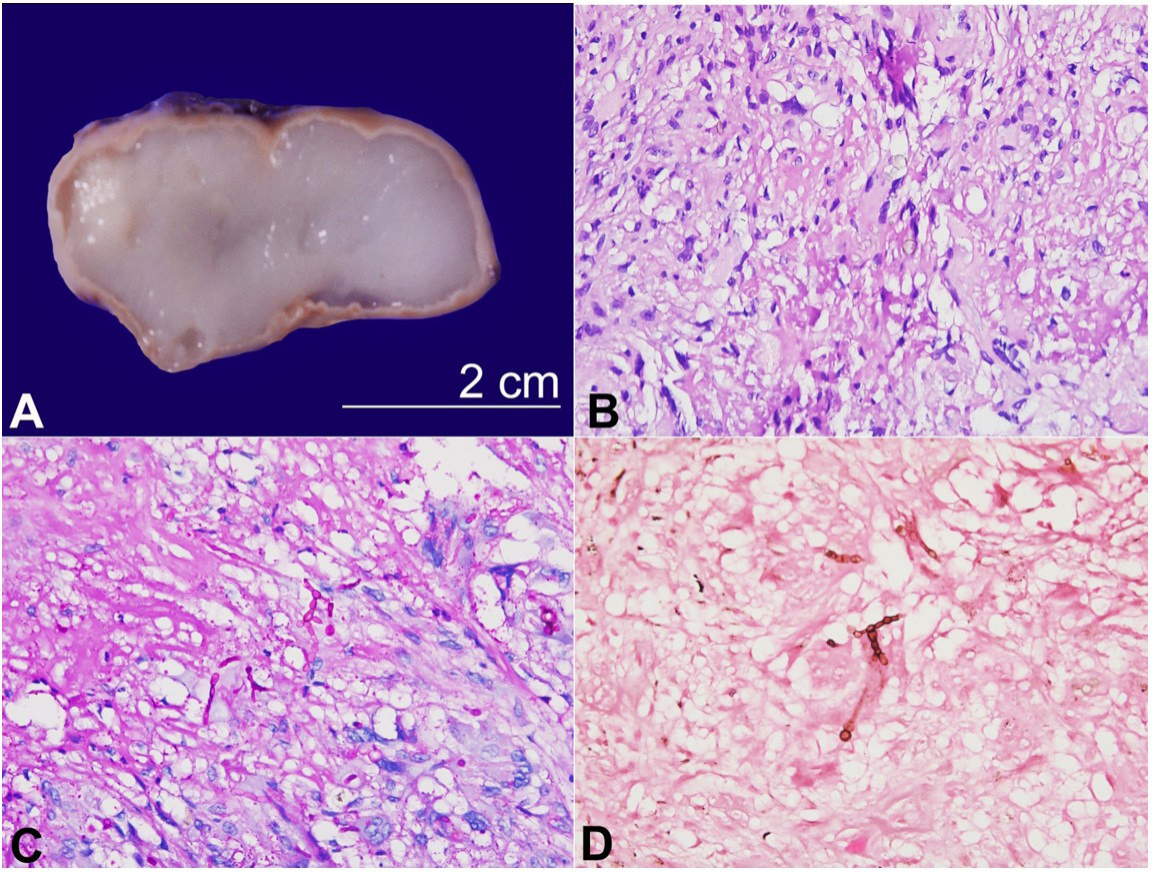
Phaeohyphomycotic cyst. **A -** A well-encapsulated and unilocular cyst measuring 4.5x2.2x1.8cm in size (scale bar = 2.5cm); The cyst lumen contains homogenous and translucent gelatinous soft material; **B -** Multinucleated giant cells showing fungal profile with septate, branching and globose swelling (H&E; x200); **C -** Periodic Acid-Schiff stain showing bright magenta positivity (PAS stain; x200); **D -** Fontana Masson stain giving brownish black color due to melanin in the fungal cell walls (x200).

Thus, the nodular swelling was wholly excised and sent for histopathological examination. Grossly, a well-encapsulated mass measuring 4.5x2.2x1.8 cm was submitted for histological analysis (Figure 1A). The external aspect appeared intact, yellowish-white, and congested. A unilocular cyst was identified on serial slicing with a capsular thickness of 0.1-0.2cm. The cut surface was soft in consistency with homogenous and gelatinous translucent material within the lumen. On light microscopy, an outer thick fibrous capsule layer was identified. Just beneath this capsular layer was the vascularized granulation tissue, proliferating fibroblasts, numerous multinucleated foreign and Langhan’s giant cells, and variable lymphoplasmacytic cell infiltrate. Also, abundant basophilic mucoid material with a background of many degenerated cells. These giant cells engulfed pigmented fungal profiles that depicted branching, septate and globose swelling (Figure 1B). Periodic Schiff-Acid stain gave bright magenta color to these fungi (Figure 1C), whereas Fontana Masson stain gave brownish black color due to melanin in the fungal cell walls (Figure 1D). Given the morphology, a diagnosis of a phaeohyphomycotic cyst was rendered. On follow-up, the patient is doing well, and has not received any antifungal agent. The leading treatment choice in non-invasive subcutaneous phaeohyphomycosis is local excision.

## References

[1] Ajello L (1986). Hyalohyphomycosis and phaeohyphomycosis: two global disease entities of public health importance. Eur J Epidemiol.

[2] Schnitzler N, Peltroche-Llacsahuanga H, Bestier N, Zündorf J, Lütticken R, Haase G (1999). Effect of melanin and carotenoids of Exophiala (Wangiella) dermatitidis on phagocytosis, oxidative burst, and killing by human neutrophils. Infect Immun.

[3] Revankar SG, Sutton DA (2010). Melanized fungi in human disease. Clin Microbiol Rev.

[4] Ben-Ami R, Lewis RE, Raad II, Kontoyiannis DP (2009). Phaeohyphomycosis in a tertiary care cancer center. Clin Infect Dis.

